# Critical Evaluation of Embedding Media for Histological Studies of Early Stages of Chick Embryo Development

**DOI:** 10.3390/mps6020038

**Published:** 2023-04-04

**Authors:** Melyssa Kmecick, Mariliza Cristine Vieira da Costa, Eduardo da Costa Ferreira, Maritana Mela Prodocimo, Claudia Feijó Ortolani-Machado

**Affiliations:** 1 Laboratory of Embryotoxicology, Department of Cell Biology, Biological Sciences Sector, Federal University of Paraná, Av. Cel. Francisco Heráclito dos Santos, 100, Curitiba 81.531-980, PR, Brazil; 2 Laboratory of Cell Toxicology, Department of Cell Biology, Biological Sciences Sector, Federal University of Paraná, Av. Cel. Francisco Heráclito dos Santos, 100, Curitiba 81.531-980, PR, Brazil

**Keywords:** chick embryo, histological processing, historesin, paraplast, polyethylene glycol

## Abstract

A histological examination is an important tool in embryology, developmental biology, and correlated areas. Despite the amount of information available about tissue embedding and different media, there is a lack of information regarding best practices for embryonic tissues. Embryonic tissues are considered fragile structures, usually small in size, and frequently challenging to position correctly in media for the subsequent histological steps. Here, we discuss the embedding media and procedures that provided us with appropriate preservation of tissue and easier orientation of embryos at early development. Fertilized *Gallus gallus* eggs were incubated for 72 h, collected, fixed, processed, and embedded with paraplast, polyethylene glycol (PEG), or historesin. These resins were compared by the precision of tissue orientation, the preview of the embryos in the blocks, microtomy, contrast in staining, preservation, average time, and cost. Paraplast and PEG did not allow correct embryo orientation, even with agar–gelatin pre-embedded samples. Additionally, structural maintenance was hindered and did not allow detailed morphological assessment, presenting tissue shrinkage and disruption. Historesin provided precise tissue orientation and excellent preservation of structures. Assessing the performance of the embedding media contributes significantly to future developmental research, optimizing the processing of embryo specimens and improving results.

## 1. Introduction

Histological studies of developing embryos have played a critical role in embryology and developmental biology fields, uncovering cellular and tissue mechanisms of morphogenesis [1,2,3] and providing detailed descriptions of the developmental features of different species [4,5]. Light microscopy of tissue sections is also an important tool in correlated fields, such as teratology. Cell and tissue shape and structure may predict function; thus, morphological examinations can identify alterations and explain dysfunctions and diseases. This makes histological observation a valuable device for a more detailed characterization of teratogenicity [6]. Due to technological advances in microscopy, it is possible to analyze microscopic images in an accurate, objective, repeatable, and quantitative manner, free from both the limits of human vision and the subjectivity of the observer, through computational microscopy [7]. Notwithstanding its importance, few studies discuss the application of histological assessment in embryology, developmental biology, and associated areas, as well as adaptations in methodologies in favor of the particularities of the embryo specimen.

The chicken (*Gallus gallus*) embryo is a funded animal model that provides a great system for studies in embryology, developmental biology, and evolutionary developmental biology (evo-devo) [8,9,10]. Additionally, it has been used for developmental toxicity studies [11,12,13]. These embryos develop quickly, are easily experimentally manipulated, and offer good cost effectiveness [14]. Furthermore, the chicken embryo development is molecularly and morphologically similar to other vertebrates, most notably during the phylotypic period [15,16,17]. However, despite these advantages, working with embryos is a challenge because of their fragility and reduced size, mostly at the early stages of chick embryos (up to 4 days of incubation). These features make it difficult to obtain proper histological sections with excellent tissue preservation and correct orientation.

The scientific literature demonstrates that several embedding media have been used for routine histology [18] of animal adult tissues and plant samples, such as paraffin-based resins. However, embryo tissues require more delicate handling and routine procedures usually do not maintain proper preservation of structure and cellular detail [19]. Embedding media such as polyethylene glycol (PEG) and glycol methacrylate (GMA)-based resins are potential alternatives for embryo histology due to their properties and processing protocols. However, there are no reports in the literature on the use of PEG for the embedding of early chick embryos. Additionally, the application of glycol-methacrylate-based resins is underexplored in this area.

Paraffin is the most common embedding medium for histology. Its usual commercially available formulations are mixtures of long-chain alkanes and plastic polymers, which melt at 56–58 °C [20]. Paraplast is a commercial resin, composed of highly purified paraffin and plastic polymers, which provides quality sections and is compatible with most routine stains and immunohistochemistry protocols, which melt at 56–57 °C [21,22]. Its formulation is known as Paraplast Plus when dimethyl sulfoxide (DMSO) is present, which offers faster infiltration times and favors sectioning (thickness down to 2–4 μm) [23]. However, due to its insolubility in water, its processing requires transitional solvents such as xylene before infiltration of the tissues [24]. This step often hardens and shrinks the tissues [25], especially those of a delicate specimen such as early chick embryos, impairing the final result.

Polyethylene glycols are polymers of ethylene oxide, produced under alkaline catalysis. They are known as “PEG” plus a numerical value, which represents its mean molecular weight [26]. PEG is a water- and alcohol-miscible embedding medium commonly used in plant histology [27] and histochemistry [28], but also applied to animal tissue histology [29,30], immunohistochemistry [30,31], enzymatic histochemistry, and histofluorescence [32]. PEG embedding allows a wide range of section thicknesses (1–150 μm) which can be obtained by varying the molecular weights of the PEGs used [33]. Nevertheless, its use as a single embedding media of animal tissues is poorly discussed and there are no recent studies on this topic. Its low acceptance happened probably because of problems during sectioning at high ambient humidity, and in mounting [33], due to PEG’s hygroscopic properties [34]. Additionally, there is no study on its applications for the histology of early stages of embryos, or immunolabeling, although its use seems promising due to the gentler processing when compared with paraffin-based resins. Among the advantages over paraplast is its solubility in water, excluding the need for dehydration for its infiltration into the tissue, in addition to a lower melting temperature.

GMA-based resins, such as historesin, are hydrophilic embedding matrixes that provide good morphological preservation because their protocols do not require clearing agents and high infiltration temperatures [35]. Several mixtures have been described and different commercial kits are available, in which the proportion of the monomer and other components may vary [36]. Embedding with GMA-based resins allows a wide range of section thicknesses (0.5–5 μm), depending on the embedding medium and knife used [36]. Thus, different levels of cell detail, contrast, and sharpness may be explored [37]. Additionally, GMA does not react with chemical groups in the tissue, which is important for staining methods. Thus, they are compatible with many histological and histochemical protocols used for paraffin sections with some modifications [38].

To date, there is little information in the literature regarding the ideal method for obtaining precise tissue orientation and optimal morphological preservation of early chick embryo sections. To fill this gap, in this work we tested three different embedding resins (paraplast, polyethylene glycol, and historesin) to find the best protocol for the histology of early stages of chicken embryos. Our findings further the knowledge and optimize the embedding methodology that applies to all areas using the chicken embryo as a model.

## 2. Materials and Methods

### 2.1. Animals

Fertilized unincubated *Gallus gallus* eggs were provided by a hatchery in Curitiba, State of Parana (PR), Brazil. A total of approximately 50 eggs were used, from at least three different batches. All procedures were approved by the Animal Use Ethics Committee from the Biological Sciences Sector of Federal University of Parana (CEUA/BIO-UFPR, certificate no 1098; http://www.bio.ufpr.br/portal/ceua/, accessed on 27 February 2023).

### 2.2. Incubation

Eggs with unbroken shells were cleaned with 70% ethanol and randomly placed in an incubator (Biochemical Oxygen Demand-BOD incubator/SL-224, SOLAB Cientifica, Piracicaba, Brazil ) with air cells facing upwards. The temperature during the incubation period (72 h) was maintained at 38 ± 0.5 °C, with 60% humidity and constant ventilation [39].

**Figure 1:**
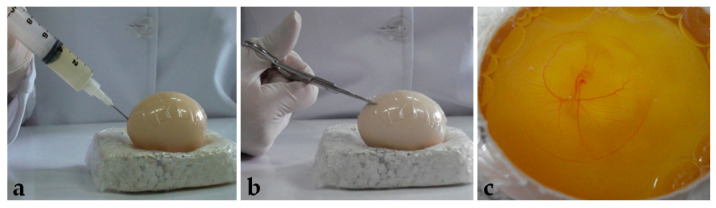
Egg opening: (**a**) Removing of albumen. (**b**) Window opening. (**c**) Embryo visualization.

**Figure 2:**
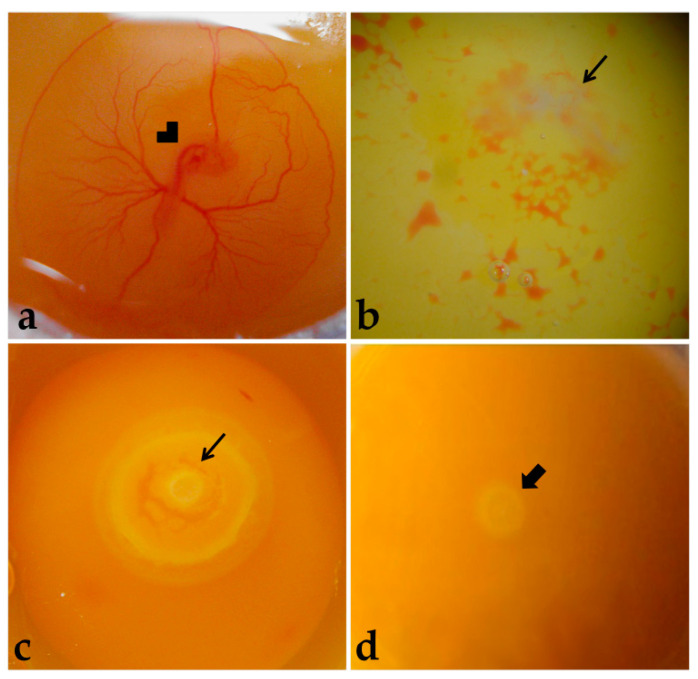
Embryo viability. (**a**) Live embryo (72 h). (**b**,**c**) Dead embryos. (**d**) Embryo classified as not having development resumed with incubation. Arrowhead: Embryo. Thinner arrow: Dead embryo. Thicker arrow: Blastoderm.

After seventy-two hours of incubation, the eggs were opened using the windowing method described by Korn and Cramer [40]. Before opening, the egg was turned 90°, so the large surface lied horizontally. After removing the egg from the incubator, adhesive tape was placed on the shell to avoid breaking. Then, 5 mL of albumen was removed with a syringe and needle (Figure 1a), and, with a scissor (Figure 1b), a window of approximately 6 cm^2^ was opened (Figure 1c) and embryo viability was determined (Figure 2).

Embryos were considered alive if they were bright rose-colored, presented heartbeats, and had intact extraembryonic blood vessels (Figure 2a). Dead embryos presented a whitish and opaque vitelline membrane, absence of heartbeats, and non-intact extraembryonic blood vessels (Figure 2b), or embryonic discs more developed than at laying, meaning that development was resumed with incubation, but was terminated before embryo harvesting (Figure 2c). Moreover, some embryos were classified as not having resumed development with incubation (Figure 2d). The dead embryos were discarded.

Live embryos were collected and transferred to a Petri dish with PBS, where their extraembryonic membranes were removed. Then, embryos were fixed in 2% paraformaldehyde (in PBS) for 72 h, in a 24-well plate, for further histological examination.

### 2.3. Embryo Orientation

**Figure 3:**
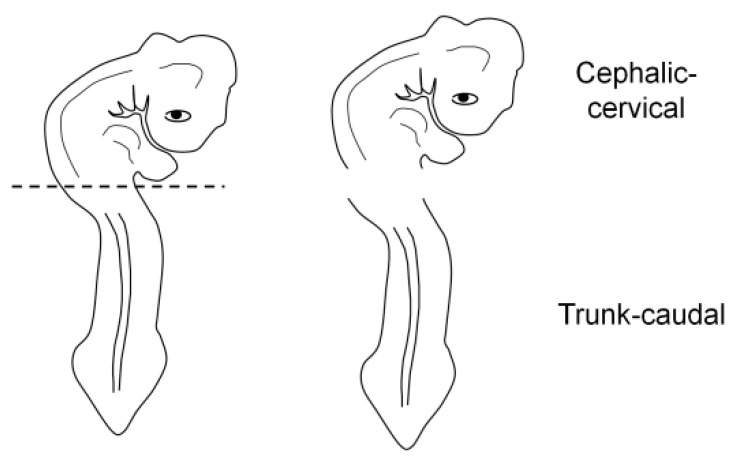
Embryo fragmentation into cephalic-cervical and trunk-caudal portions.

For the correct orientation of the embryo, specimens were embedded as either a whole embryo or a fragment (cephalic-cervical and trunk-caudal region) (Figure 3). This strategy was adopted due to difficulties at the moment of orientation, as the heavier head tends to sink and elevate the trunk, generating an undesired angle. To obtain the fragments, embryos were separated into regions with a blade, under a stereomicroscope (SZ40, Olympus, Shinjuku, Japan).

### 2.4. Paraplast

Fixed embryos were washed in PBS for 15 min and dehydrated in 70–95% ethanol, followed by two changes of 100% ethanol, 10–15 min each. The embryos were then cleared in xylene (two changes, 3–5 min each), and infiltrated in Paraplast Plus (Leica Biosystems, Wetzlar, Germany) at 58 °C (three baths, 15–60 min each) [41]. Then, they were embedded in paraplast and oriented for obtaining transversal sections. Embedding was performed in metallic molds with covering cassettes. Each block contained a whole embryo or an embryo fragment.

Sections of 5 μm were obtained in a semiautomatic microtome (RM 2145, Leica Biosystems, Wetzlar, Germany), using histological disposable steel blades, and placed on glass slides covered with albumin and water. After the distention of the sections on a heated plate, the slides were dried overnight at room temperature. Then, slides were placed on xylene for removing the resin, hydrated in ethanol (100-70%, 3–6 min each) and distilled water, and stained with Harri’s hematoxylin (30 s) and eosin-floxin (30 s) (H&E). Next, sections were dehydrated in ethanol (95% and twice in 100%, 1–3 min each) and ethanol-xylene (1:1—3 min), cleared in xylene (twice for 3 min), and mounted with Permount^TM^ (Thermo Fisher Scientific, Waltham, EUA) and coverslip. All procedures are detailed in Table 1 and Table 2.

As it was difficult to obtain the precise embryo orientation for sectioning by direct incorporation into paraplast, we chose to perform a pre-embedding tissue immobilization in an agar–gelatin solution before paraplast embedding. This protocol was based on the studies of Buzzel [42], Ghassemifar and Franzén [43], Jones and Calabresi [44], and McClelland et al. [45], with modifications. Stock solutions of agar (4%) (Sigma-Aldrich, San Luis, EUA) and gelatin (Sigma-Aldrich, San Luis, EUA) (5%) in PBS were prepared to obtain a final solution of agar–gelatin (2 or 4%—2.5 or 5%). This solution was optimized, and the final concentrations chosen were agar at 2% and gelatin at 5%.

**Figure 4:**
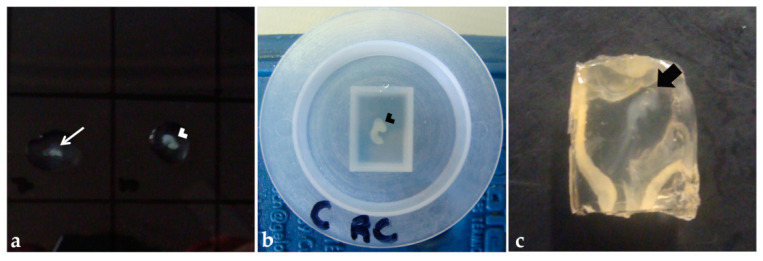
Pre-embedding in agar–gelatin solution: (**a**) Embryo fragments into a drop of solution on a Petri dish. (**b**) Cephalic and cervical regions of an embryo in a polyethylene mold filled with the solution. (**c**) Agar–gelatin block after polymerization. Thinner arrow: Trunk-caudal region. Arrowhead: Cephalic and cervical region. Thicker arrow: Embryo.

Fixed embryos were washed in PBS (twice, for 5 min each), then transferred to the agar–gelatin solution on a drop of the solution on a Petri dish (Figure 4a) [45] or a polyethylene mold filled with the solution (Figure 4b), and oriented for obtaining transversal sections. After polymerization, the blocks were removed from the molds (Figure 4c), trimmed, and the anterior portion of the block was stained with aqueous eosin 1% (30 s). Then, the blocks were placed into identified histological cassettes and stored in ethanol 70% or NaCl 0.9% solution, for at least 24 h. Paraplast processing was performed as described previously in this section, except for the step with xylene that was replaced for amyl acetate in the processing of some blocks, to test which clarification solution would bring better results.

### 2.5. Polyethylene Glycol

In PEG 1500 (Synth) processing, two methods were tested, with dehydration (method 1) (M1) and without (method 2) (M2), as described by Wolosewick [46]. First, fixed embryos were washed in PBS (15 min) and then processed with methods 1 or 2. In processing M1 with ethanol and PEG, the embryos were dehydrated in ethanol (25–100%, 10 min each) and infiltrated in two solutions of PEG and 100% ethanol, in the proportions 1:1 and 2:1, for 30 or 60 min each. Then, samples were transferred to 100% PEG (two changes), for 30 or 60 min (Table 3).

In PEG-only processing (M2), solutions were prepared in distilled water in concentrations (*v*/*v*) of 25, 50, and 70%. Embryos were infiltrated in these solutions for 15 to 30 min each. Then, they were transferred to 100% PEG (two changes, 15 or 30 min each) (Table 4).

All processing was performed in a 24-well microplate (histological cassettes can be used as well). At all steps with solutions containing PEG, samples were maintained at 55 °C. After infiltration with PEG, embryos were embedded in paper molds. On a heated plate, the identified mold was filled with PEG and the embryo was properly oriented for obtaining transversal sections. This enables higher polymerization time, especially on cold days, allowing more time for tissue manipulation. Each block contained a whole embryo or an embryo fragment. The blocks were kept in a container with silica, at room temperature or 4 °C for overnight polymerization. Afterward, the blocks were removed from the molds and stored in a container with silica, to avoid humidification and softening. Then, before sectioning, they were trimmed and fixed on wooden supports with previously melted PEG.

Using disposable blades, 5 μm cross sections were produced in a semiautomatic microtome (Leica RM 2145, Leica Biosystems, Wetzlar, Germany). The sections were placed on albumin or chrome gelatin-coated (0.1% or 1%) slides, with or without Triton^TM^ X-100 (Sigma-Aldrich, San Luis, EUA) solution (0.1% in PBS). After the distention of sections on a heated plate, slides were dried overnight at room temperature. Then, histological sections were hydrated in 70% ethanol and distilled water and stained with Harri’s hematoxylin (30 s) and eosin-floxin (30 s) (Table 5). After drying, the sections were covered with Permount^TM^ (Thermo Fisher Scientific, Waltham, EUA, Waltham, MA, USA) and coverslip.

### 2.6. Historesin

**Figure 5:**
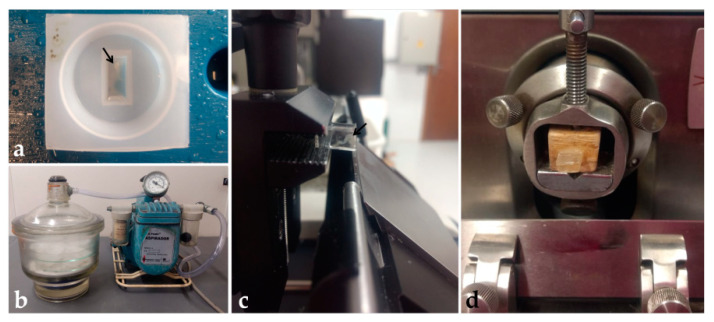
Historesin processing: (**a**) Embryo embedded into the polyethylene mold. (**b**) Desiccator and vacuum pump. (**c**) Block placed in the semi-automatic microtome showing the side view of the transparent block and the blade. (**d**) Block fixed on wood and positioned in the manual microtome. Black arrow: Embryo.

This protocol was based on González Santander et al. [19], with modifications. The working solution was prepared according to the Historesin Embedding Kit (Leica Biosystems, Wetzlar, Germany) by diluting 5 g of the activator compound in 50 mL of resin (hydroxyethyl methacrylate) and storing at 4 °C. In a 24-well plate, embryos were washed in PBS for 2 h, and dehydrated in ethanol (30%—5 min; 50–90%—10 min; twice in 100%—10 min each). Then, tissues were immersed in pre-infiltration solution (ethanol 100% and working solution, 1:1) for 2 h, and infiltration solution (working solution) for at least 12 h, both at room temperature. Next, each embryo was placed in 600 μL of embedding solution (working solution and hardener compound, 15:1) (Table 6) in polyethylene molds and orientated to obtain transversal sections (Figure 5a). To maintain the embryo at the desired position, a cold pack and a heating plate were used to control the initial polymerization time, and tweezers or a wooden pick to hold the embryo in position. Each block was identified and placed in a vacuum desiccator with silica for 48 h for complete polymerization (Figure 5b). Then, blocks were removed from the molds and stored in a container with silica, to avoid humidification and softening.

For tissue sectioning, two microtomes were used: a semi-automatic one (Leica RM 2145) and a manual one (Spencer 820, Vernon Hills, EUA), to evaluate the best method for holding the block. In the semi-automatic microtome, the block was directly fixed to the support and sectioned with tungsten knives (Figure 5c). In the manual microtome, the block was fixed to a wooden support with cyanoacrylate glue before being positioned on the microtome and sectioned with non-disposable steel knives (Figure 5d). Blocks were trimmed until the tissue reached the sectioning surface. Toluidine blue (0.1%) staining was used to confirm the presence of the sample in the sections. After confirmation, sections of 5 μm were obtained, placed on drops of water on clean glass slides, distended, and dried on a heated plate. Then, sections were hydrated in distilled water (3 min) and stained with Harri’s hematoxylin (1–30 min), and aqueous eosin 1% (1–15 min) (Table 7). After drying, sections were covered with Permount^TM^ (Thermo Fisher Scientific, Waltham, EUA) and coverslip.

### 2.7. Analysis

All histological slides were analyzed under a light microscope (Metrimpex Hungary/PZO-Labimex, Studar lab). About 900 slides, including 250 slides of paraplast-only, 150 slides of pre-embedded specimens, 250 slides of PEG samples, and 250 slides of historesin samples. Additionally, about 10,000 sections were analyzed in total. Selected sections were documented under a bright-field photomicroscope (Olympus BX40, DP71 Camera 12.5 megapixels, DPController software, Olympus, Shinjuku, Japan).

The parameters evaluated for considering the best method for embedding early chick embryos were (1) precision of tissue orientation, (2) the preview of the embryos in the blocks, (3) microtomy, (4) contrast in staining, (5) preservation of structures (morphology and presence of artifacts), (6) time, and (7) cost of processing.

Following the examination of the slides, the three embedding methods tested were assigned a score for each evaluated parameter, using an evaluation index developed specifically for this study. The scores ranged from 0 to 5 and are presented in Table 8. The analysis was qualitative, and the slides were assessed by three observers.

The precision of tissue orientation was categorized from impossible (score = 0) to excellent (score = 5). The scoring of this parameter was comparative between the resins. The assessment of embryo visualization in the blocks was categorized as impossible (score = 0) or possible (score = 1). Microtomy was ranked from impossible (score = 0) to good (score = 3) based on the time required for sectioning a whole embryo and the quality of resulting sections. The scoring of this parameter was also comparative between the resins. As for the contrast in staining, this was considered weak (score = 0) to excellent (score = 5). Here, the differentiation of cytoplasm, nucleus, nucleolus, and mitotic figures was considered. The tissue preservation was categorized by the maintenance of morphology, which was classified from awful (score = 0) to excellent (score = 5), and the presence of artifacts, from highly frequent (score = 0) to not observed (score = 5). In this parameter, the shape and integrity of embryonic cells and structures (ectoderm, neural tube, notochord, somite, and mesoderm) were considered. The methods with the longer time and higher cost received a lower score (score = 0) and the others were scored until the maximum (score = 5), based on their percentage from the higher time or cost.

## 3. Results

### 3.1. Embryo Orientation

#### 3.1.1. Paraplast

**Figure 6:**
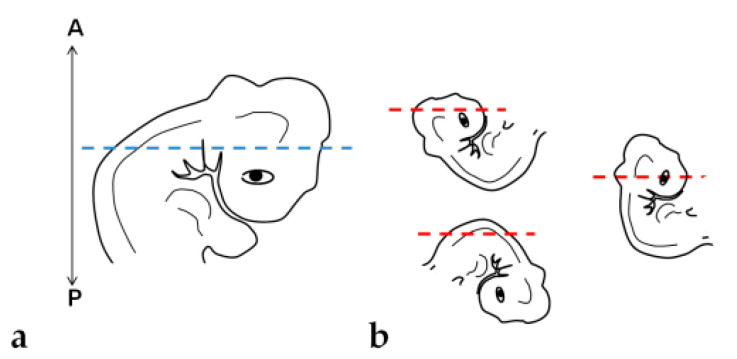
Embryo positioning in the block: (**a**) Desired orientation and plane of section (blue dashed line). (**b**) Orientations and planes of section (red dashed lines) obtained. A: Anterior portion of the block. P: Posterior portion.

**Figure 7:**
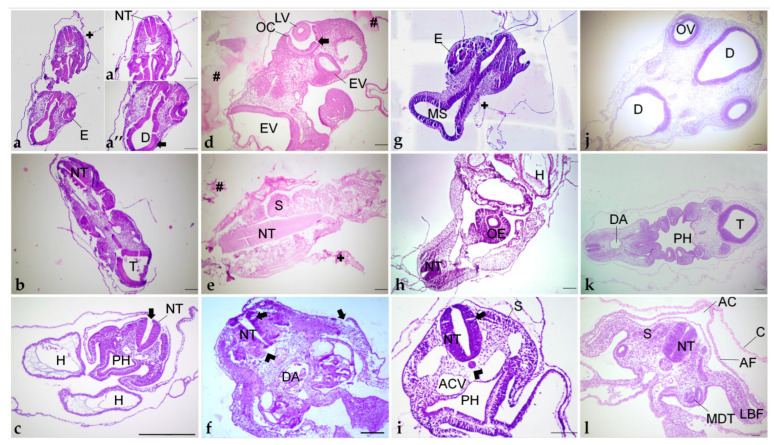
Different orientations of histological sections obtained of embryos embedded in paraplast (**a**–**c**); paraplast with pre-embedding in agar–gelatin (**d**–**f**); PEG (**g**–**i**); and historesin (**j**–**l**). (**a**,**d**,**g**,**j**) Cephalic region. (**b**,**e**,**h**,**k**) Cervical region. (**c**,**f**,**i**,**l**) Trunk region. (**a’**): Cervical portion. (**a”**): Cephalic portion. AC: Amniotic cavity. ACV: Anterior cardinal vein. AF: Amniotic fold. C: Chorion. D: Diencephalon. DA: Dorsal aorta. E: Eye. EV: Encephalic vesicle. H: Heart. LBF: Lateral body folds. LV: Lens vesicle. MDT: Mesonephric duct and tubule. MS: Mesencephalon. NT: Neural tube. OC: Optic cup. OV: Optic vesicle. OE: Oesophagus. PH: Pharynx. S: Somite. T: Telencephalon. +: Detachment. #: Agar–gelatin residue. Arrowhead: Shrinkage. Arrow: Disruption. Staining: H&E. Scale bar = 100 μm.

Tissue orientation was difficult to perform when using paraplast as an embedding medium (Figure 6a,b). Paraplast processing steps caused alteration in the embryo, turning it rigid and opaque, impairing the identification of the embryo’s body axes. Additionally, due to the quick polymerization of the resin at room temperature and the reduced size of embryos (approximately 5 mm), it was not possible to place and maintain them in the correct position to obtain transversal sections of cephalic, cervical, trunk, and caudal regions. After dividing the embryo into two parts, cross sections in the desired plane of section were obtained of trunk-caudal regions (Figure 7c), but not of cephalic-cervical regions (Figure 7a,b).

Pre-embedding in agar was performed before the paraplast embedding to improve embryo orientation. The first attempt with a pre-embedding solution of 2% agar and 2.5% was not efficient because the drops of agar–gelatin dissolved in the storage solutions (NaCl 0.9% or ethanol 70%), and the blocks prepared on the polyethylene molds dissolved in the 0.9% NaCl solution. Posterior clarification with xylene dissolved these blocks, and amyl acetate made them opaque, and it was impossible to see embryo orientation. In trying to prevent the block from dissolving in xylene, the concentration of agar and gelatin was increased to 4% and 5%, respectively, but due to the formation of lumps, it was not used. Thus, agar concentration was reduced to 2%, with gelatin maintained at 5%, and these blocks resisted xylene clarification but also became non-transparent. To overcome this matter and enable embryo orientation, the anterior portion of the block was stained with eosin. However, the color vanished during processing. Thus, with this method, embryos were not visible for positioning, and the embedding was performed without visual guidance. By chance, incorrect (Figure 7d,e) and correct (Figure 7f) orientation of the embryos occurred, of cephalic-cervical and trunk-caudal portions, respectively.

#### 3.1.2. Polyethylene Glycol

PEG embedding was then tested, seeking a more precise tissue orientation. This method offered more time to place and maintain the tissue in the desired position, as the polymerization time at room temperature is higher than paraplast. However, the achieved quality of orientation was similar to paraplast, with the cephalic-cervical regions (Figure 7g,h) being more difficult to orientate than trunk-caudal regions (Figure 7i).

#### 3.1.3. Historesin

This method was the most suitable for the samples. Due to its translucency and lengthy polymerization, there was enough time to place and hold the small-sized embryos correctly at the desired position. Additionally, the processing steps caused the embryo to become more transparent, while it was still possible to identify the embryo’s body axes. Due to the correct orientation, embryo structures were easily identified (Figure 7j–l).

### 3.2. Preview of the Embryos in the Blocks, Tissue Sectioning (Microtomy), and Staining

#### 3.2.1. Paraplast

**Figure 8:**
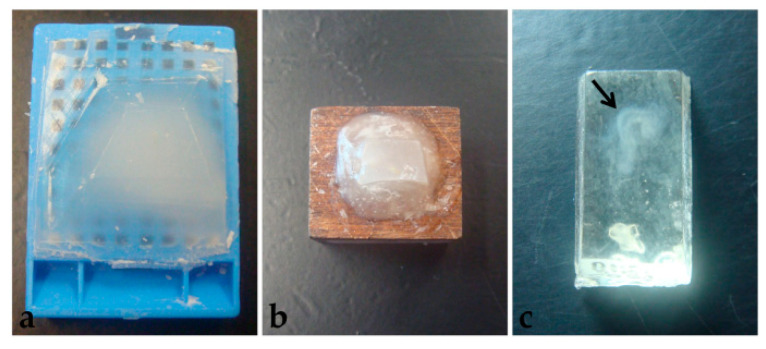
Polymerized blocks. (**a**) Paraplast. (**b**) PEG. (**c**) Historesin. Arrow: Embryo.

Another difficulty observed in paraplast processing, with or without pre-embedding, was that embryos were not visible in the blocks (Figure 8a). Therefore, several specimens were lost in microtomy since the presence of tissue in the sections was not easily identified.

**Figure 9:**
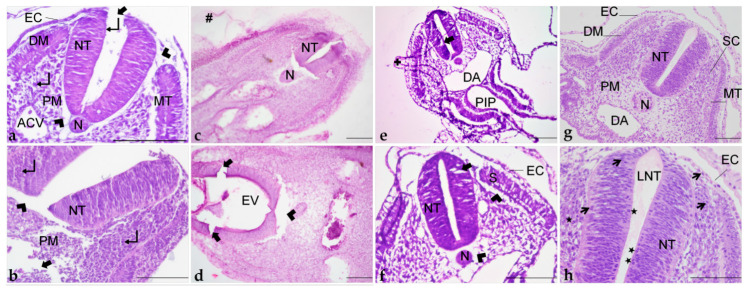
Histological sections of embryos embedded in paraplast (**a**,**b**); paraplast with pre-embedding in agar–gelatin (**c**,**d**); PEG (**e**,**f**); and historesin (**g**,**h**). ACV: Anterior cardinal vein. DA: Dorsal aorta. DM: Dermatome. EC: Ectoderm. EV: Encephalic vesicle. LNT: Lumen of the neural tube. MT: Myotome. N: Notochord. NT: Neural tube. PM: Paraxial mesoderm. PIP: Posterior intestinal portal. S: Somite. SC: Sclerotome. +: Detachment. #: Agar–gelatin residue. ★: Mitotic figures. ↵: Nucleus. Arrowhead: Shrinkage. Thicker arrow: Disruption. Thinner arrow: Evident nucleus and nucleolus. Staining: H&E. Scale bar = 100 μm.

H&E staining provided an appropriate contrast between the nucleus and cytoplasm. It was possible to observe the cellular cytoplasm, nucleus, and with micrometric adjustment, the nucleolus, in most of the sections (Figure 9a,b).

Microtomy of pre-embedded blocks did not provide suitable tissue sections, as they were fragmented by the blade, impairing histological examination (data not shown). Dehydration and infiltration times were adjusted to overcome the brittleness of the tissue, but it did not improve the integrity of the sections. Additionally, inadequate staining was frequently observed (Figure 7d,e). In general, it was not possible to distinguish the cellular cytoplasm, nucleus, and nucleolus (Figure 9c,d), even with adjustment of the staining protocol.

#### 3.2.2. Polyethylene Glycol

Microtomy of PEG blocks was challenging because sections were very delicate, as well as the tissue, and crumbling was frequent. Additionally, similar to what happened in paraplast processing, it was not possible to preview the embryos in the blocks (Figure 8b), which led to the loss of specimen material. Moreover, blocks were stored at room temperature, with desiccant silica, to avoid humidification, which also compromised microtomy. Section distension was only satisfactory in Triton X-100 0.1% solution, which also provided good adhesion to the 1% gelatin-coated slides. Nevertheless, during staining, all sections placed over albumin-covered slides and 0.1% gelatin detached.

H&E staining did not result in good contrast, as cytoplasm, nucleus, and nucleolus were not easily differentiated (Figure 9e,f).

#### 3.2.3. Historesin

Unlike the other two techniques described here, historesin enabled embryo visualization in the block (Figure 8c). It was necessary to fix the blocks to the wooden support the day before the microtomy, to allow the glue to dry completely. Additionally, to avoid softening of the resin, which would compromise the microtomy process, blocks fixed to the support were stored in a container with silica until sectioning. Microtomy is more difficult and time-consuming than the other methods tested, due to the hardness of the resin. Additionally, the formation of ribbons is rare.

H&E staining offered excellent contrast and great cellular detail, with cytoplasm, nucleus, and nucleolus easily distinguished (Figure 9g,h). The staining protocol time was also longer than in the other resins but presented fewer steps. The optimal time was 30 min in Harri’s hematoxylin and 12 min in eosin. These times may vary due to staining solution conditions (e.g., age of the solution, frequency of use) [47]. It is important to reinforce that staining solutions cannot contain alcohol, since it would cause the sections to detach from the slide.

### 3.3. Tissue Preservation Quality

#### 3.3.1. Paraplast

Embryo processing in paraplast provided proper tissue preservation (Figure 9a,b). Somite structure was preserved, with identifiable dermatome and myotome (Figure 9a). Nevertheless, artifacts that may compromise a detailed morphological assessment were observed. Tissue shrinkage was frequently noticed around the neural tube and at the paraxial mesoderm (Figure 9a,b). Additionally, tissue disruption occurred, mainly at the ectoderm, neural tube, and mesenchyme (Figure 9a,b).

The agar/gelatin pre-embedding step compromised tissue preservation (Figure 9c,d), causing tissue crumbling and retraction (Figure 9d), frequent agar–gelatin residues (Figure 9a), as well as poor staining (Figure 9c,d).

#### 3.3.2. Polyethylene Glycol

Two different methods of infiltration with PEG were tested, but no difference was observed in terms of preservation. Tissue shrinkage (Figure 9g) was similar to paraplast, but cellular morphology was considered improper as in most sections it was not possible to differentiate cytoplasm, nucleus, and nucleolus (Figure 9f,g). Additionally, neural tube cells were not juxtaposed, and spaces between them were very commonly observed (Figure 9f,g). Beyond that, more delicate portions of the sections, as extraembryonic membranes, detached and folded over the tissue (Figure 9f).

#### 3.3.3. Historesin

Despite the fragility of early chick embryos, this resin provided excellent tissue preservation, with the maintenance of morphology (Figure 9g,h) and minimal shrinkage when compared to the other resins. Additionally, cellular detail was superior to the other methods tested with distinctly observed cytoplasm, nucleus, and nucleolus (Figure 9h). Additionally, the surface ectoderm was observed as a single-layered cuboidal epithelium (Figure 9h), a feature not seen clearly in the other preparations. The neural tube was preserved as a thick pseudostratified epithelium, with juxtaposed cells, presented with optimal morphology (Figure 9g,h). It was even possible to identify mitotic figures in the neuroepithelium, next to the lumen of the neural tube, and in the sclerotome (Figure 9h).

### 3.4. Average Block Preparation and Cost

The average time used in the preparation of the block with each resin was noted as it can also be a decisive factor when choosing embedding resins. Preparation with paraplast lasted about 6 h, but when the pre-embedding step with agar–gelatin was used, this time was raised to 30 h because a series of additional procedures were needed. The shortest time between the three resins was for PEG, in M1 and M2, which lasted 3 and 4 h, respectively. Meanwhile, the time of processing for historesin embedding was similar to that of paraplast with agar–gelatin pre-embedding (30 h), because the specimen infiltration with this resin is slower.

The cost of historesin kits and the blades required for microtomy are quite high. On the other hand, the cost of using paraplast and PEG is low, being approximately 10 and 5% of the value of historesin, respectively.

Table 9 summarizes and compares the parameters evaluated in each resin, through scoring. A higher score indicates a better outcome. All results were worse in the processing in which pre-blocking in agar–gelatin was used and, therefore, were not included in the comparison.

## 4. Discussion

Due to the fragility and reduced size of early chick embryos (up to 4 days of incubation), structural preservation is lost with histological routine processing. Information about the ideal method for this developmental period is still limited. Thus, in this study, we demonstrated how different embedding resins preserve morphological features, such as embryonic structure and cellular details of chick embryo sections.

Tissue orientation is crucial for the demonstration of proper morphology, correct identification of structures, and diagnosis of possible damage [48,49,50], particularly in samples that present different organizations depending on the plane of section, such as embryos. Among the tested resins, paraplast and PEG did not offer appropriate conditions to position the embryo in the mold, due to its quick polymerization which depends only on temperature. Thus, paraplast and PEG embedding require an oven and a heated plate, or embedding station equipment to perform tissue embedding, due to their high melting points. In contrast, historesin allowed embryo positioning in the mold, because it presents a slow polymerization rate at room temperature. This process is dependent on a catalyst (hardener), which reacts in the presence of the activator and generates free radicals that act as polymerization initiators [51]. The speed of this reaction depends on temperature, which is very high and may turn the block overly hard and brittle, or, if too low, excessively soft [52]. The ideal conditions for the polymerization of glycol methacrylate (GMA)-based resins, such as historesin, vary greatly according to their composition, such as temperature (from −20 °C to 40 °C), and, in some cases, the need for ultraviolet light and microwaves [36]. However, there are no studies that describe these conditions for historesin. Another favorable point of historesin was its transparency after polymerization, allowing excellent visualization of the embryo in the block, which facilitates positioning during microtomy. Therefore, it is also used for other small species [53,54] or organs [55,56]. Despite having different polymerization speeds at room temperature, higher when compared with glycol methacrylate resins, PEG and paraplast allowed similar orientation quality for fragments of the trunk-caudal region.

The pre-embedding in agar–gelatin was found to be an accessible alternative in trying to obtain optimal embryo orientation with paraplast. However, it was not efficient in the processing of our samples. Even after adjustments in the dehydration, clarification, and infiltration times, it did not improve tissue preservation. This fact was not expected, because according to Jones and Calabresi [44], pre-embedding preparations should not damage tissue processing, as the agar–gelatin solution does not infiltrate into the sample [42]. Moreover, the use of an agar–gelatin solution should have prevented issues observed when they are used individually, such as sections detaching from slides and poor staining [44], as was observed in the present study.

Histology is a useful tool in embryology, developmental biology [3], and correlated fields, including developmental toxicology [22] and teratology [57]. Thus, it is clear that the maintenance of morphology is essential to obtain reliable results. During histological processing, embedding matrixes are used to impregnate the tissue, such as paraplast-, PEG-, and GMA-based resins. These matrixes provide support and allow thin sections to be obtained [21]. As preservation status is the result of the interaction of the specimen with the embedding medium, this parameter will be discussed per resin.

Paraplast did not keep appropriate preservation of chick embryo tissue, and artifacts were frequent. Its tissue processing method requires the use of intermediate solvents because it is not directly soluble in ethanol. This, together with the high temperature needed for infiltration, damaged the fragile samples, and generated artifacts due to tissue hardening and distortion [58]. These artifacts were accentuated in pre-embedded samples, because the agar–gelatin coat impaired impregnation, which had to be prolonged, resulting in higher shrinkage and crumble. Smith and Warfield [59] suggested that tissue shrinkage can occur due to the effect of heat on collagen. To reduce these temperature artifacts, Paraplast X-tra could be an alternative, as it has a lower melting point (53–54 °C) [60]. PEG melts at a similar temperature (55 °C) but also did not yield satisfactory structural preservation.

The use of PEG 1500 did not yield appropriate tissue structural preservation in either method tested. Additionally, method 1 (with dehydration) required about 1 h less than method 2 (without dehydration) to obtain the same section quality. Nevertheless, our work is the first to approach PEG as an embedding medium for early chick embryos and to compare its use with other resins. Despite the low quality of morphology preservation observed in this study, this medium is recommended for immunohistochemical techniques due to the high antigenicity of the tissue sections obtained, including those of soft materials such as early chick embryos [61]. Additionally, PEG is proper for immunofluorescent labeling, and its preservation of cellular detail is described as superior to frozen sections [62]. Thus, our data on PEG embedding enrich the limited literature on this matter and may help researchers in exploring its properties in immunodetection techniques.

Acrylic resins are appropriate for embedding fragile, soft, and temperature-sensitive tissues such as embryos, because infiltration and polymerization occur at room temperature, minimizing protein degradation and damage to cellular structures [36]. Indeed, historesin yielded the best preparation of the embryo sections and high microscopic image resolution, which, according to González Santander et al. [19], is close to the in vivo state. Furthermore, its hydrophilic properties exclude the need for highly toxic clearing agents [63], such as xylene, which hardens and shrinks the tissue [25].

The adhesion of the tissue sections to the slides is crucial to avoid detachment during immunohistochemistry or staining procedures. In some cases, properly cleaned and dried slides are enough. GMA sections attach to slides by heat and water evaporation on a heated plate [64]. However, paraplast and PEG sections require adhesive coating on slides, such as albumin, gelatin, poly-L-lysine, or 3-aminopropyltriethoxysilane [65]. Albumin is commonly used with paraplast sectioning, in which adhesion is promoted by heating the slides to 55–60 °C, after placing the sections. This process coagulates the albumin layer, making it insoluble in water and preventing the sections from detaching [66]. The use of chrome gelatin coating provides charge to the slides, allowing them to attract negatively charged tissue sections [67]. This method was the most efficient in holding PEG sections to the slides, with a 1% gelatin solution, which provided more cross-linked bonds due to a higher concentration of gelatin. Additionally, Triton X-100 solution increased the adhesion of the sections to the slides, as described by Smithson et al. [33].

Histological staining is used to enhance tissue features and promote contrast between structures [68]. Hematoxylins stain nuclear chromatin and other acidic cellular elements [58]. Among them, Harris’ hematoxylin is the most commonly used combined with eosin [69] to demonstrate the general histological structure of tissues. In contrast, eosin stains cell cytoplasm and most connective tissue in varying tones and intensities of pink, orange, and red. Among the types of eosins available, eosin Y is the most used and is soluble in ethanol and water [47], which makes it suitable for GMA processing. Additionally, floxin is often used to provide a darker cytoplasmic color [58]. In this study, different levels of color and contrast were obtained on the sections of early chick embryos with H&E staining. The nuclear staining was noticeable in the sections of all the used methods, except in those of pre-embedded embryos. On the other hand, eosin staining was less evident in PEG sections. According to Bancroft and Layton [47], variations in staining may occur due to factors such as fixation, variations in processing steps, section thickness, and excessive hot plate temperatures. Additionally, our results point out that the embedding medium is a factor related to processing that also affects color tones and contrast provided by dyes.

Although only historesin provided excellent tissue preparation for morphological assessment of 72 h chick embryos, the three tested resins have their advantages and disadvantages, depending on the purpose of the study. Thus, when choosing which embedding medium to apply it is important to consider factors such as morphological preservation and antigenicity. Paraplast and PEG are recommended for immunohistochemistry approaches, without the need for precise morphological preservation, and for histological examination of later stages of embryo development, which are less fragile than early stages. GMA-based resins provide precise structure preservation and also allow immunostaining techniques [70]. Another relevant difference between these three resins is the time of processing. Paraplast and PEG present similar processing times, whereas that of historesin is close to paraplast with pre-embedded samples. Beyond processing, the tissue staining protocol of glycol methacrylate resins is longer as the medium is not previously removed from the sections and slows stain penetration [71]. However, fewer steps are necessary because ethanol and xylene are not used. In terms of cost, paraplast and PEG resins have a similar price and require the usual equipment of a histology laboratory (stove, embedding station, cassettes, steel blades). In contrast, the historesin kit has a higher cost, as well as the blades, such as tungsten and glass knives, required for sectioning [36].

## 5. Conclusions

Historesin yielded the best preparation of early chick embryos, combining morphological preservation and precise tissue orientation. In addition to excellent structural preservation, embryo tissues embedded in historesin presented fewer artifacts and higher cellular detail when compared to paraplast- and PEG-embedded samples. This is the first study to compare these three embedding methods for early chick embryo histology. Thus, our data highlight the best method to obtain meaningful histological assessment in developmental studies using the chick embryo as a model.

## Figures and Tables

**Table 1:** Summary of procedures in paraplast processing.

Solution	Time Combinations (min)			
	A	B *	C	D
PBS	15	15	15	15
70% ethanol	10	15	15	15
80% ethanol	10	15	15	15
90% ethanol	10	15	15	15
95% ethanol	10	15	15	15
100% ethanol I	10	15	15	15
100% ethanol II	10	15	15	15
Xylene I	3	3	5	5
Xylene II	3	3	5	5
Paraplast I	15	15	30	60
Paraplast II	15	15	30	60
Paraplast III	15	15	30	60

* Times which had better results.

**Table 2:** H&E staining of sections in paraplast.

Solution	Time
Xylene	6 min
100% ethanol	6 min
95% ethanol	6 min
90% ethanol	3 min
80% ethanol	3 min
70% ethanol	3 min
Distilled water	Immersion, twice
Hematoxylin	30 s
Running tap water	10 min
Distilled water	Immersion, twice
Eosin-Floxin	30 s
Distilled water	Immersion, twice
95% ethanol	1 min
100% ethanol I	3 min
100% ethanol II	3 min
100% ethanol:Xylene (1:1)	3 min
Xylene I	3 min
Xylene II	3 min

**Table 3:** Summary of method 1 in PEG processing.

Solution	Time Combinations (min)	
	A *	B
PBS	15	15
25% ethanol	10	10
50% ethanol	10	10
70% ethanol	10	10
95% ethanol I	10	10
95% ethanol II	10	10
100% ethanol I	10	10
100% ethanol:PEG (1:1)	30	60
100% ethanol:PEG (1:2)	30	60
100% PEG I	30	60
100% PEG II	30	60

* Times which had better results.

**Table 4:** Summary of method 2 in PEG processing.

Solution	Time Combinations (min)	
	A	B *
PBS	15	30
25% PEG	15	30
50% PEG	15	30
70% PEG	15	30
100% PEG I	15	30
100% PEG II	15	30

* Times which had better results.

**Table 5:** H&E staining of sections in PEG.

Solution	Time
70% ethanol	3 min
Distilled water	Immersion, once
Hematoxylin	30 s
Tap water	10 min
Distilled water	Immersion, once
Eosin-Floxin	30 s
Distilled water	Immersion, once

**Table 6:** Summary of procedures in historesin processing.

Solution	Time
30% ethanol	5 min
50% ethanol	10 min
70% ethanol	10 min
80% ethanol	10 min
90% ethanol	10 min
100% ethanol I	10 min
100% ethanol II	10 min
Pre-infiltration	2 h
Infiltration	12 h
Embedding (working solution + hardener)	12 h (or until complete polymerization)

**Table 7:** H&E staining of sections in historesin.

Solution	Time Combinations (min)					
	A	B	C	D	E	F *
Distilled water	3	3	3	3	3	3
Hematoxylin	1	5	10	15	20	30
Running tap water	10	10	10	10	10	10
Distilled water	Immersion, three times					
Aqueous eosin (1%)	1	5	10	15	12	12
Distilled water I ^#^	Immersion, three times					
Distilled water II ^#^	Immersion, three times					

* Times which had better results. ^#^ Change water between sets of slides stained.

**Table 8:** Evaluated parameters with corresponding classification and description.

Parameter	Score					
	0	1	2	3	4	5
Precision of tissue orientation	Impossible	Poor	Difficult	Good	Very good	Excellent
		Short time to orientate specimen	Longer time to orientate the specimen	Longer time to orientate with some visualization of the specimen	Longer time to orientate with good visualization of the specimen	Longer and controllable time to orientate and excellent visualization of the specimen
Preview of the embryos in the blocks	Impossible	Possible	-	-		-
Microtomy	Impossible	Very difficult	Difficult	Good	-	-
		Poor quality of sections macroscopically, slower microtomy, and fewer usable sections	Poor quality of sections macroscopically and slower microtomy	Good quality of sections macroscopically and slower microtomy		
Contrast in staining	Weak	Poor	Satisfactory	Good	Very good	Excellent
	Impossible to differentiate basophilic and eosinophilic regions	Weak color differentiation between cytoplasm and nucleus	Clear color differentiation between cytoplasm and nucleus	Clear color differentiation between cytoplasm and nucleus, nucleolus evident	Clear color differentiation between cytoplasm and nucleus, nucleolus evident, different tones of color between structures	Clear color differentiation between cytoplasm and nucleus, nucleolus evident, different tones of color between structures, mitotic figures visible
Preservation						
Maintenance of morphology	Awful	Poor	Satisfactory	Good	Very good	Excellent
	All structures with compromised morphology	Four structures with compromised morphology	Three structures with compromised morphology	Two structures with compromised morphology	One structure with compromised morphology	All structures with optimal morphology
Presence of artifacts	Highly frequent	Frequent	Occasional	Rare	Very rare	Not observed
	Section with the occurrence of generalized artifacts	Up to ten artifacts/section	Up to five artifacts/section	Up to two artifacts/section	One artifact/section	Zero artifacts
Time of processing	Longer time (30 h)	80%	60%	50%	±20% (6 h)	±10% (3 h)
Cost	Higher price	±80%	±50%	±25%	±10%	±5%

**Table 9:** Comparison of scores assigned to the parameters analyzed in each resin.

Parameter	Resins		
	Paraplast	PEG	Historesin
Precision of tissue orientation	1	2	5
Preview of the embryos in the blocks	0	0	1
Microtomy	3	1	2
Contrast in staining	4	1	5
Preservation			
Morphology	3	1	5
Presence of artifacts	1	0	4
Time of processing	4	5	0
Cost	4	5	0
Total	20	15	22

## Data Availability

Not applicable.
